# Supplementary material for: High-Dose Acetaminophen with N-acetylcysteine Rescue Inhibits M2 Polarization of Tumor-Associated Macrophages
Source: Cancers (Basel). 2023 Sep 28;15(19):4770. doi: 10.3390/cancers15194770 (PMC10571846; doi:10.3390/cancers15194770)
Supplement: Supplementary file 1 [file cancers-15-04770-s001.zip › cancers-2604837-supplementary/cancers-2604837-SI.pptx]

## Slide 1
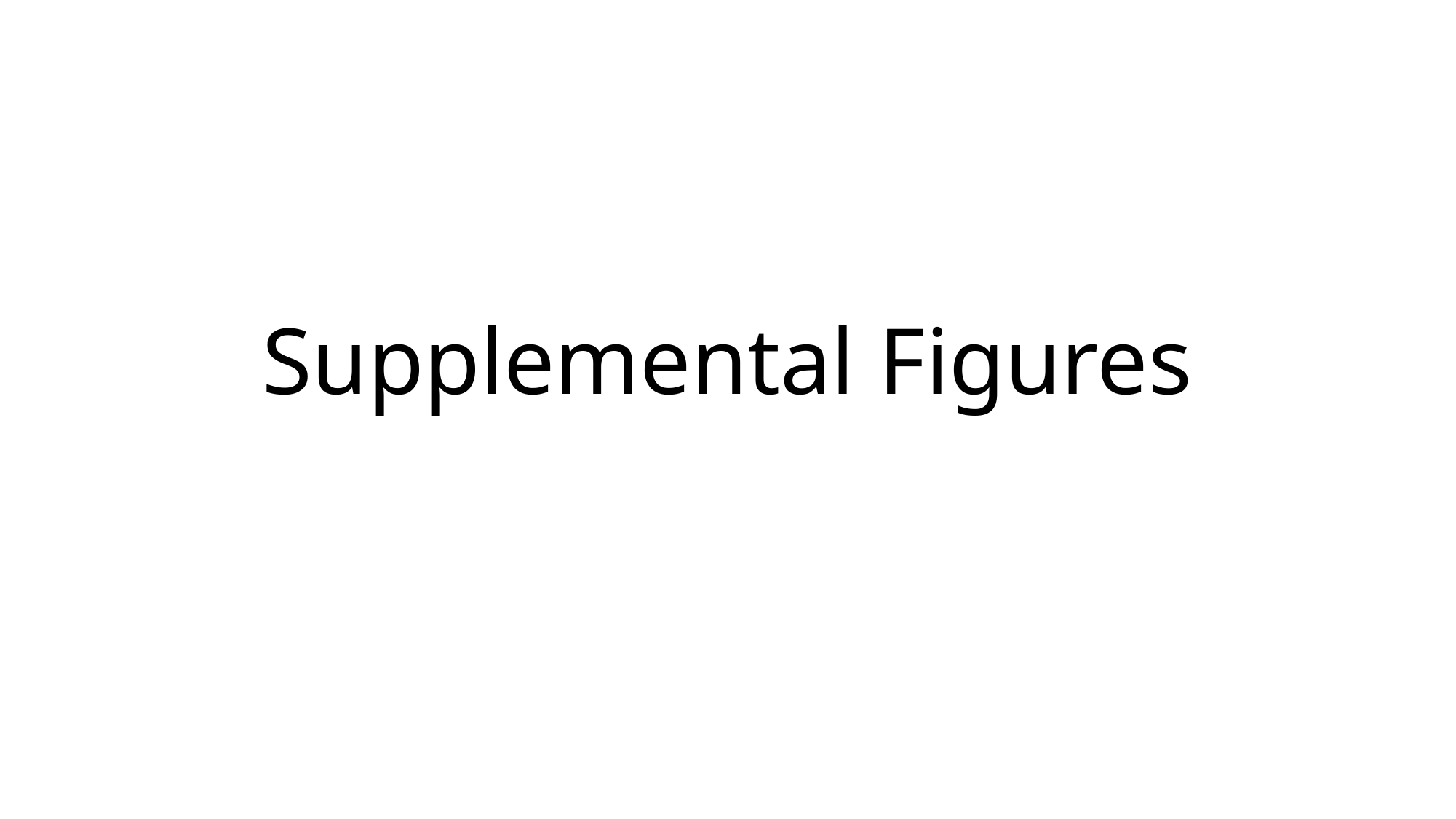

# Supplemental Figures

## Slide 2
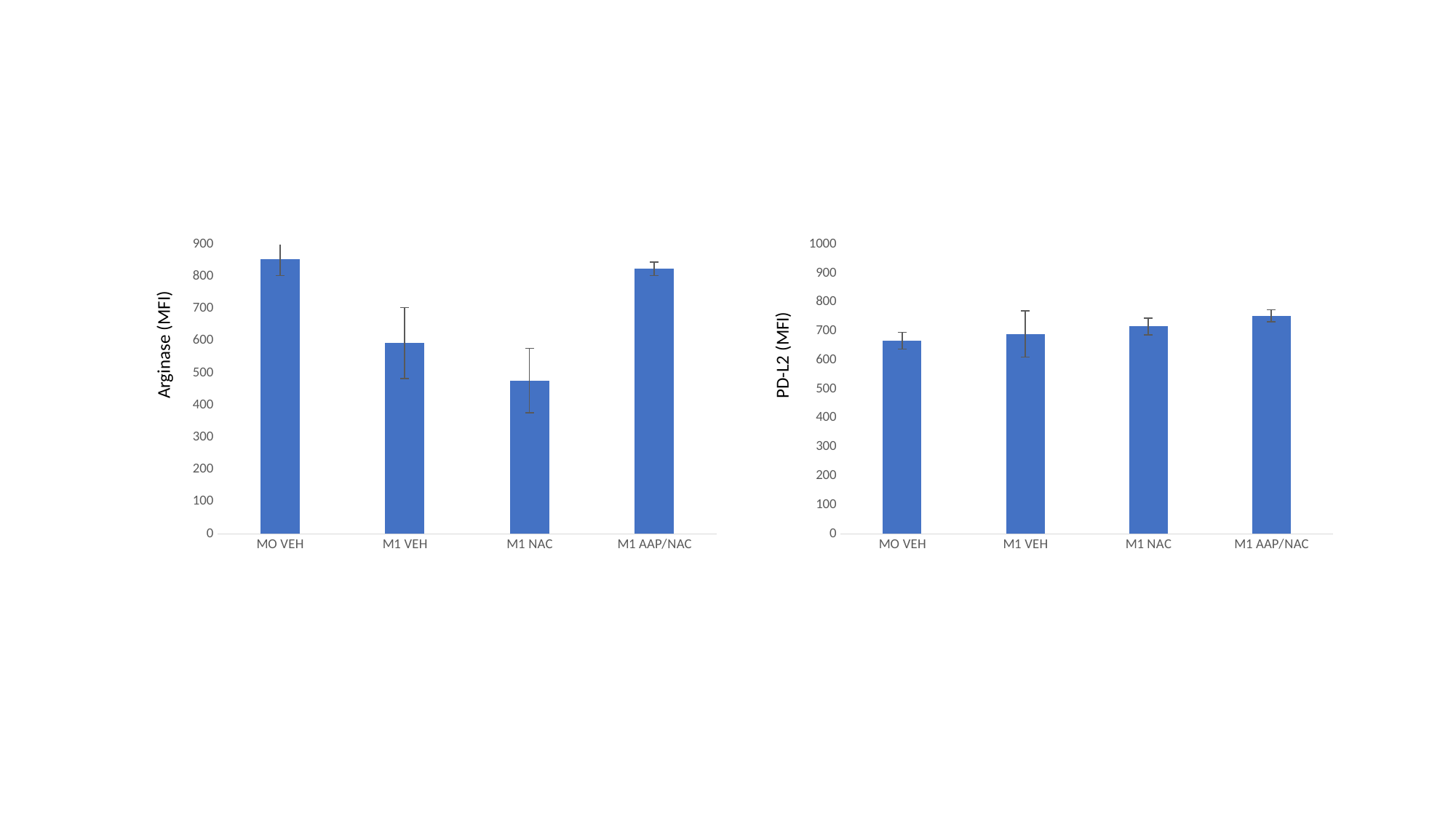

### Chart
| Category | |
|---|---|
| MO VEH | 853.3333333333334 |
| M1 VEH | 593.3333333333334 |
| M1 NAC | 476.6666666666667 |
| M1 AAP/NAC | 823.3333333333334 |
### Chart
| Category | |
|---|---|
| MO VEH | 666.6666666666666 |
| M1 VEH | 690.6666666666666 |
| M1 NAC | 716.6666666666666 |
| M1 AAP/NAC | 753.3333333333334 |Arginase (MFI)
PD-L2 (MFI)

## Slide 3
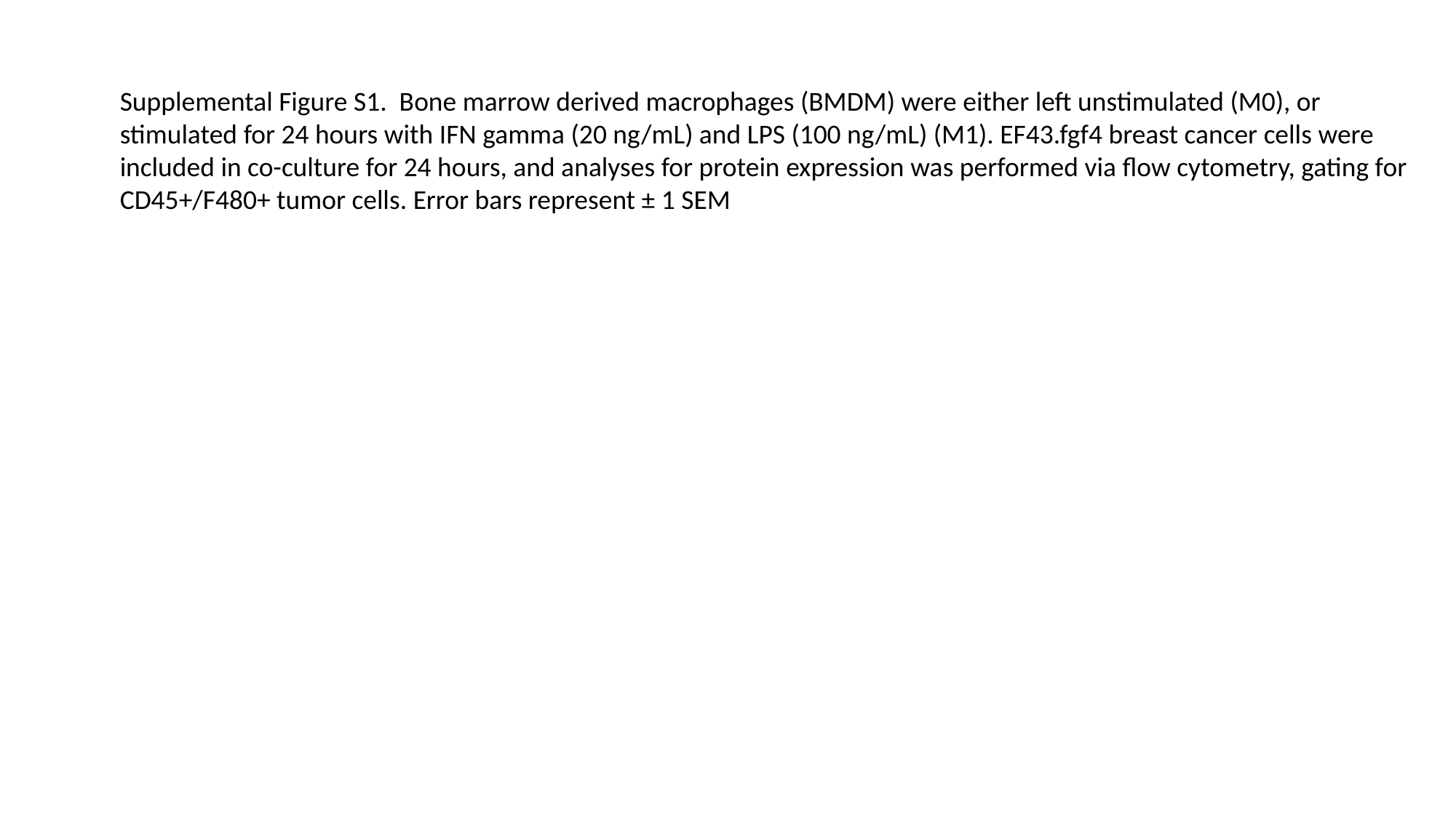

Supplemental Figure S1. Bone marrow derived macrophages (BMDM) were either left unstimulated (M0), or stimulated for 24 hours with IFN gamma (20 ng/mL) and LPS (100 ng/mL) (M1). EF43.fgf4 breast cancer cells were included in co-culture for 24 hours, and analyses for protein expression was performed via flow cytometry, gating for CD45+/F480+ tumor cells. Error bars represent ± 1 SEM

## Slide 4
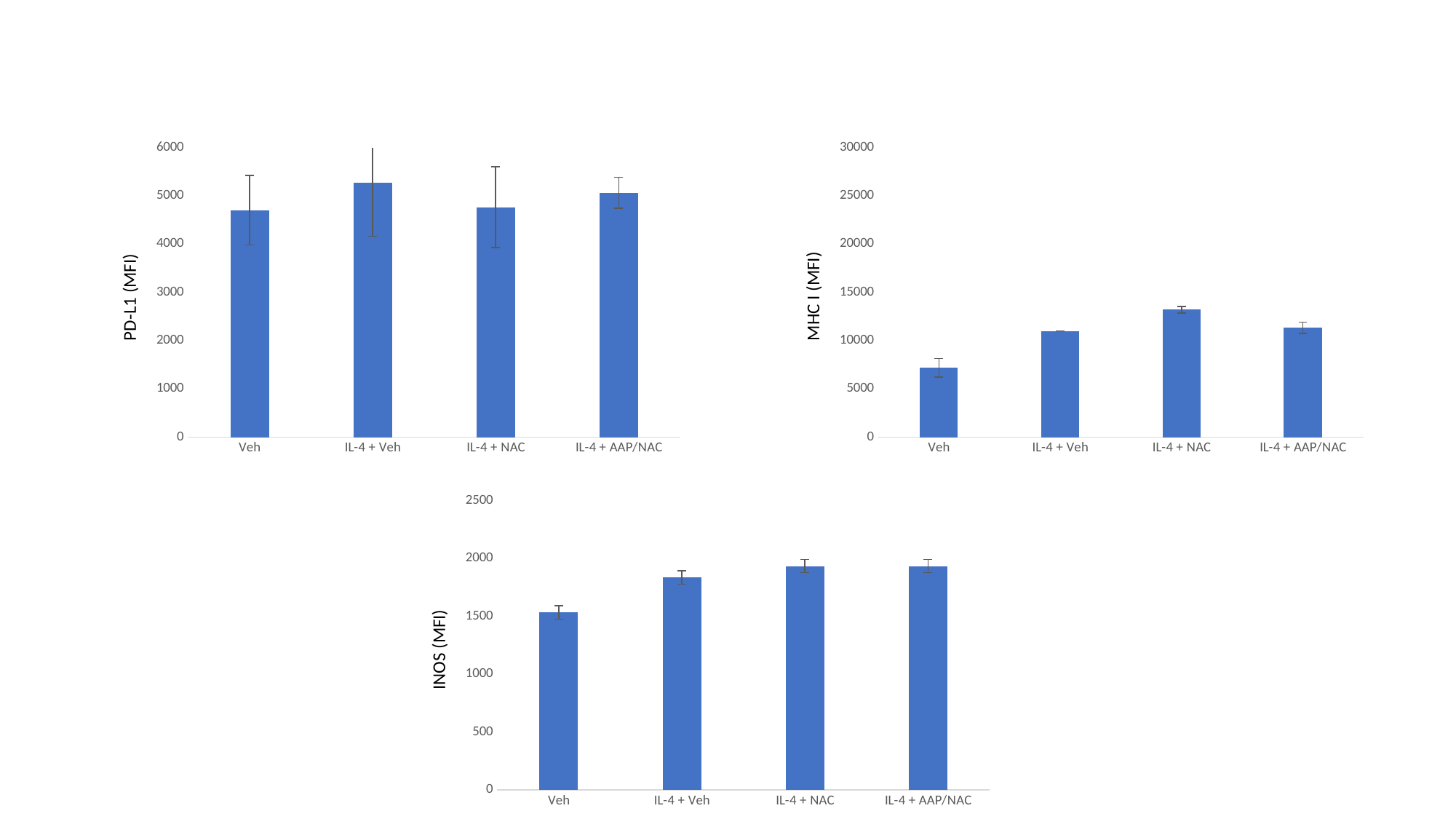

### Chart
| Category | |
|---|---|
| Veh | 4700.0 |
| IL-4 + Veh | 5266.666666666667 |
| IL-4 + NAC | 4766.666666666667 |
| IL-4 + AAP/NAC | 5066.666666666667 |
### Chart
| Category | |
|---|---|
| Veh | 7200.0 |
| IL-4 + Veh | 11000.0 |
| IL-4 + NAC | 13200.0 |
| IL-4 + AAP/NAC | 11333.333333333334 |PD-L1 (MFI)
MHC I (MFI)
### Chart
| Category | |
|---|---|
| Veh | 1533.3333333333333 |
| IL-4 + Veh | 1833.3333333333333 |
| IL-4 + NAC | 1933.3333333333333 |
| IL-4 + AAP/NAC | 1933.3333333333333 |INOS (MFI)

## Slide 5
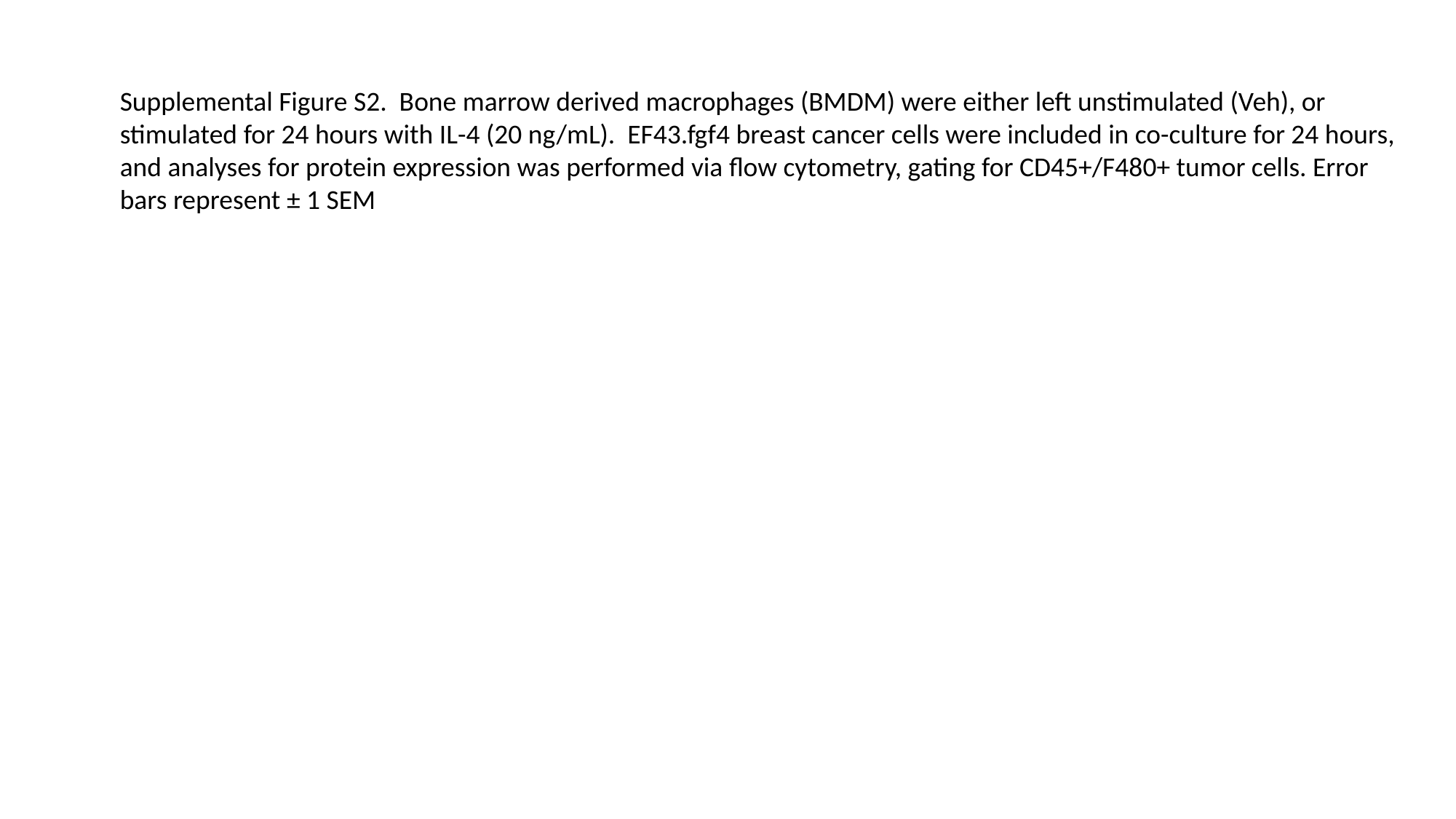

Supplemental Figure S2. Bone marrow derived macrophages (BMDM) were either left unstimulated (Veh), or stimulated for 24 hours with IL-4 (20 ng/mL). EF43.fgf4 breast cancer cells were included in co-culture for 24 hours, and analyses for protein expression was performed via flow cytometry, gating for CD45+/F480+ tumor cells. Error bars represent ± 1 SEM

## Slide 6
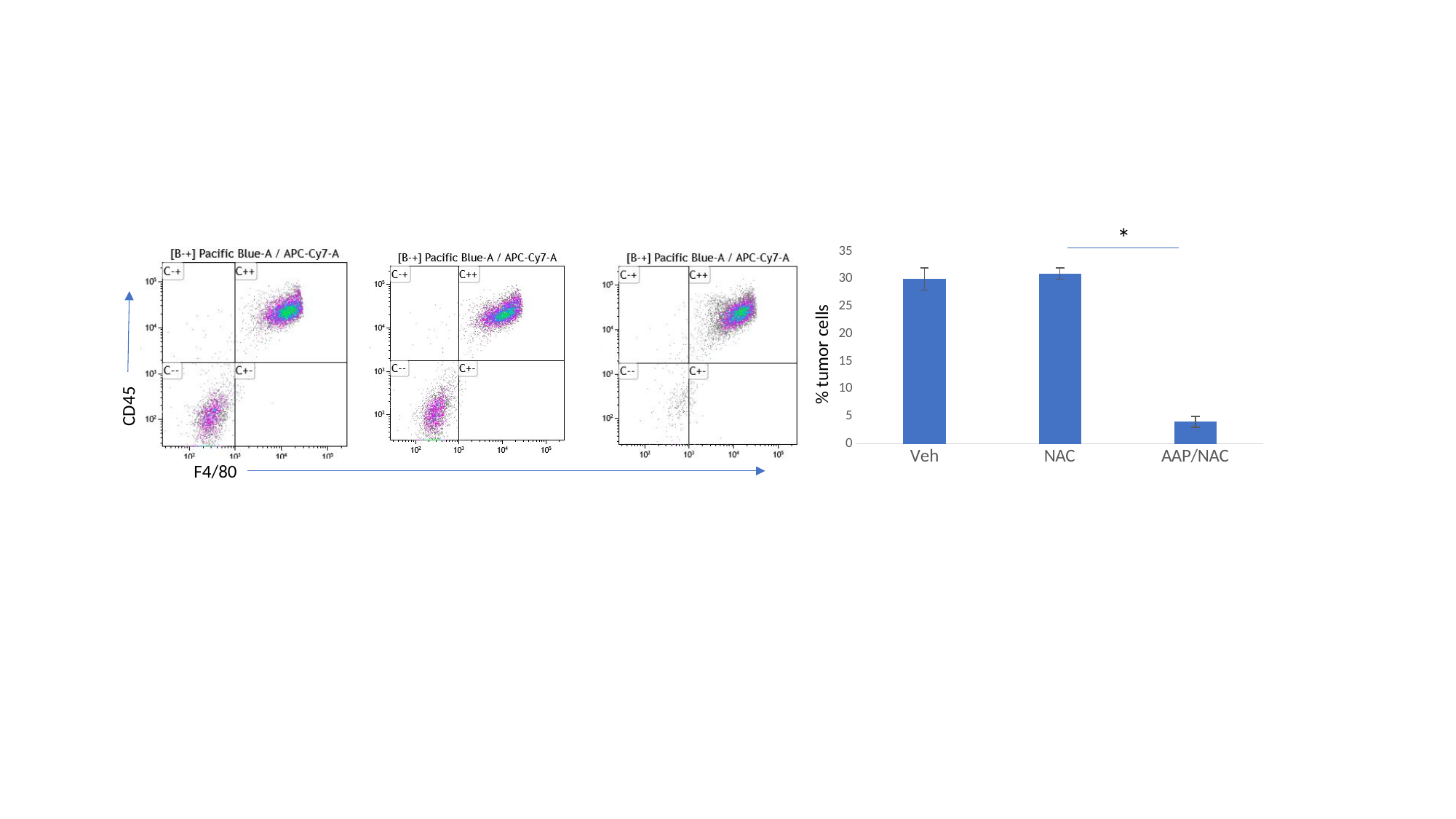

*
### Chart
| Category | |
|---|---|
| Veh | 30.0 |
| NAC | 31.0 |
| AAP/NAC | 4.0 |
% tumor cells
CD45
F4/80

## Slide 7
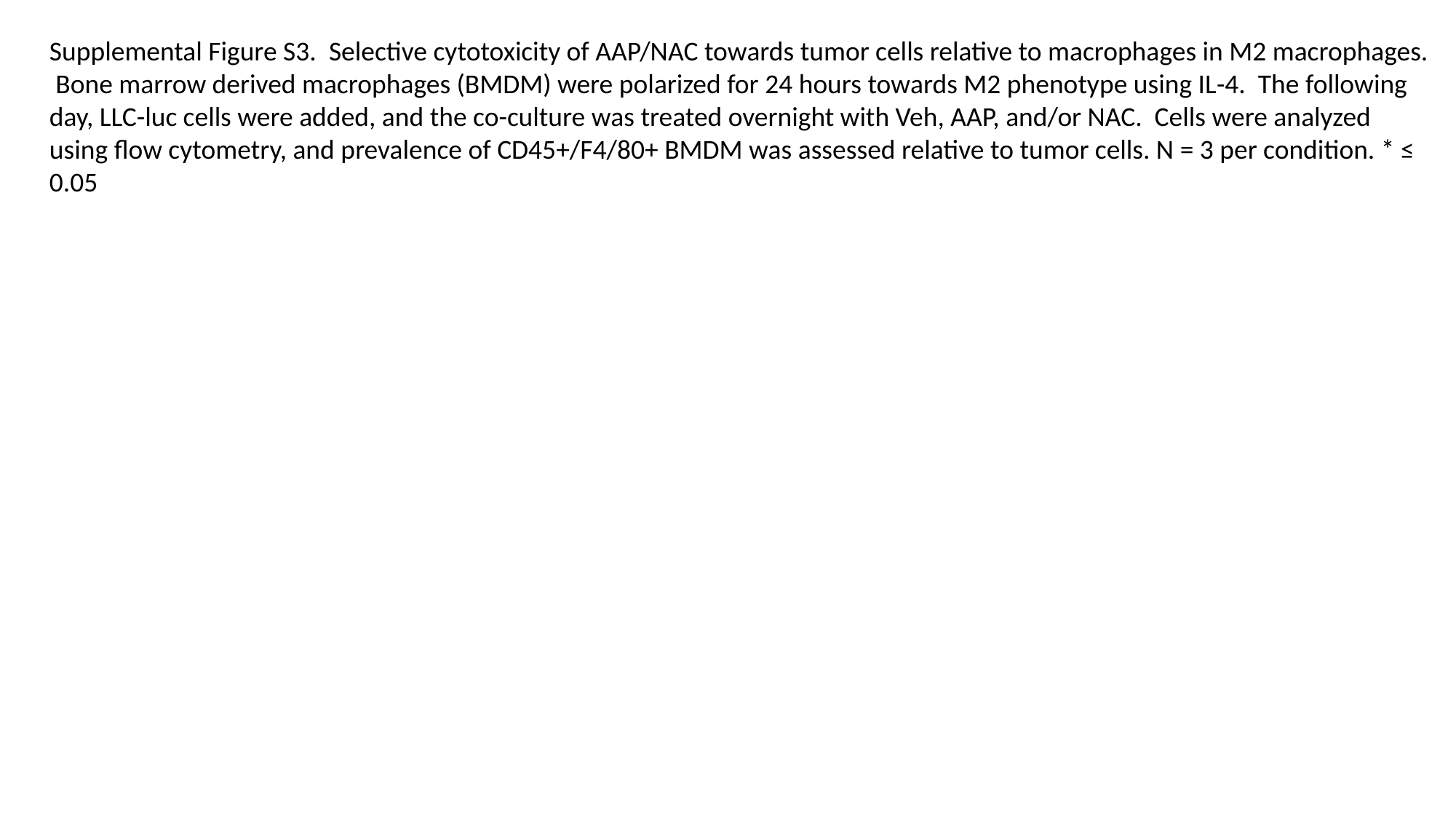

Supplemental Figure S3. Selective cytotoxicity of AAP/NAC towards tumor cells relative to macrophages in M2 macrophages. Bone marrow derived macrophages (BMDM) were polarized for 24 hours towards M2 phenotype using IL-4. The following day, LLC-luc cells were added, and the co-culture was treated overnight with Veh, AAP, and/or NAC. Cells were analyzed using flow cytometry, and prevalence of CD45+/F4/80+ BMDM was assessed relative to tumor cells. N = 3 per condition. * ≤ 0.05

## Slide 8
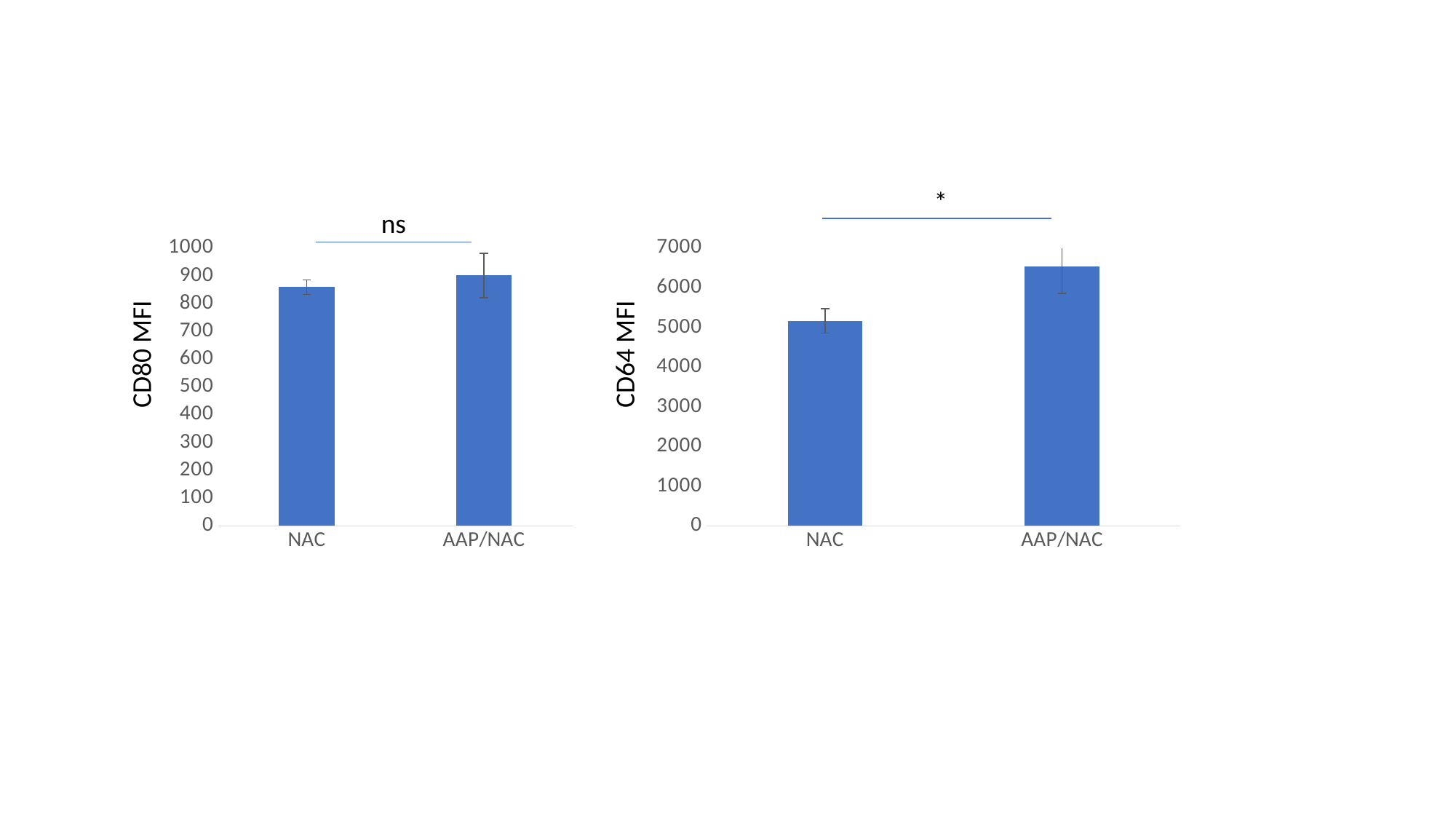

*
ns
### Chart
| Category | |
|---|---|
| NAC | 860.0 |
| AAP/NAC | 902.0 |
### Chart
| Category | |
|---|---|
| NAC | 5166.666666666667 |
| AAP/NAC | 6533.333333333333 |CD80 MFI
CD64 MFI

## Slide 9
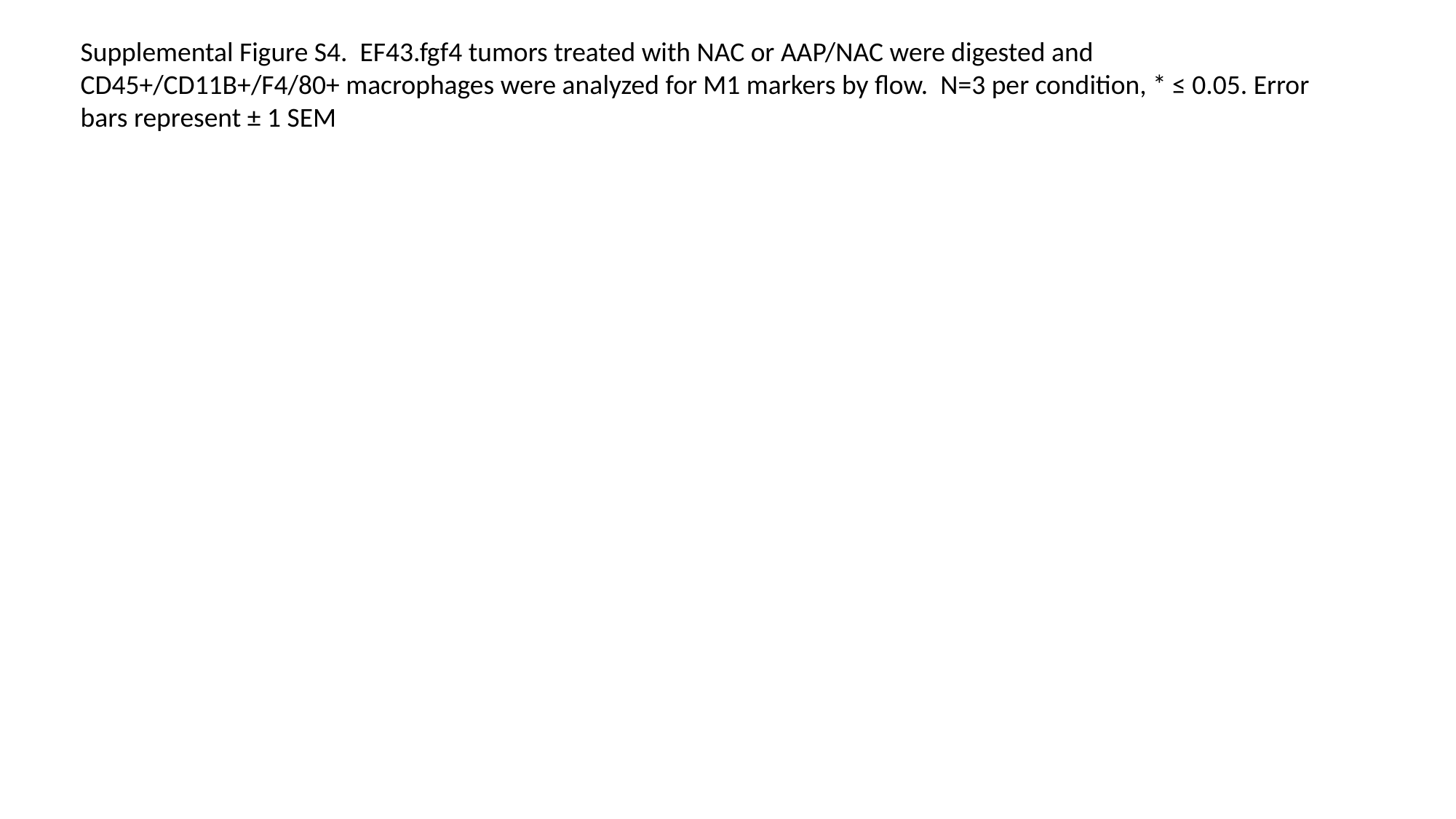

Supplemental Figure S4. EF43.fgf4 tumors treated with NAC or AAP/NAC were digested and CD45+/CD11B+/F4/80+ macrophages were analyzed for M1 markers by flow. N=3 per condition, * ≤ 0.05. Error bars represent ± 1 SEM

## Slide 10
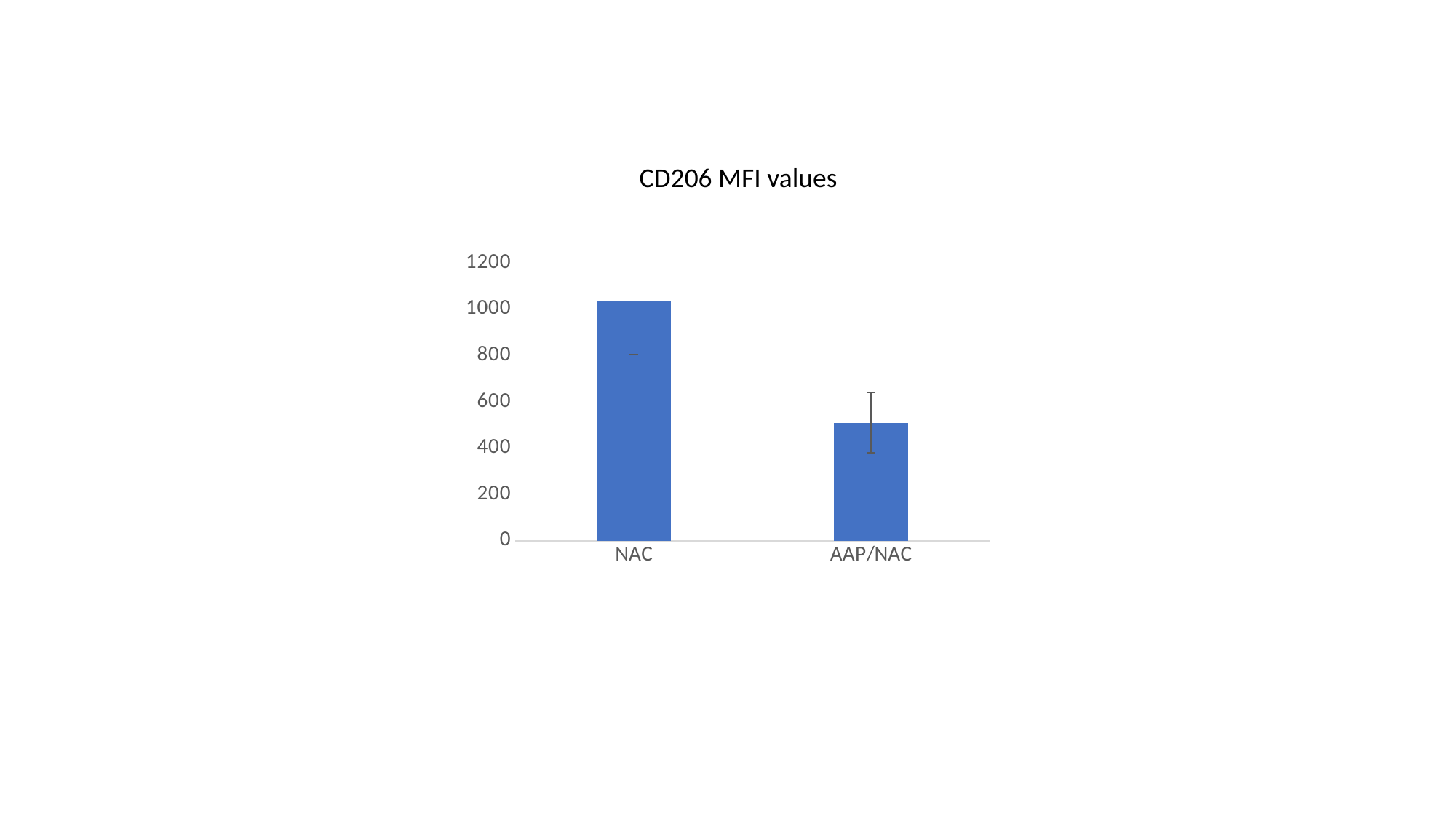

CD206 MFI values
### Chart
| Category | |
|---|---|
| NAC | 1035.0 |
| AAP/NAC | 510.0 |

## Slide 11
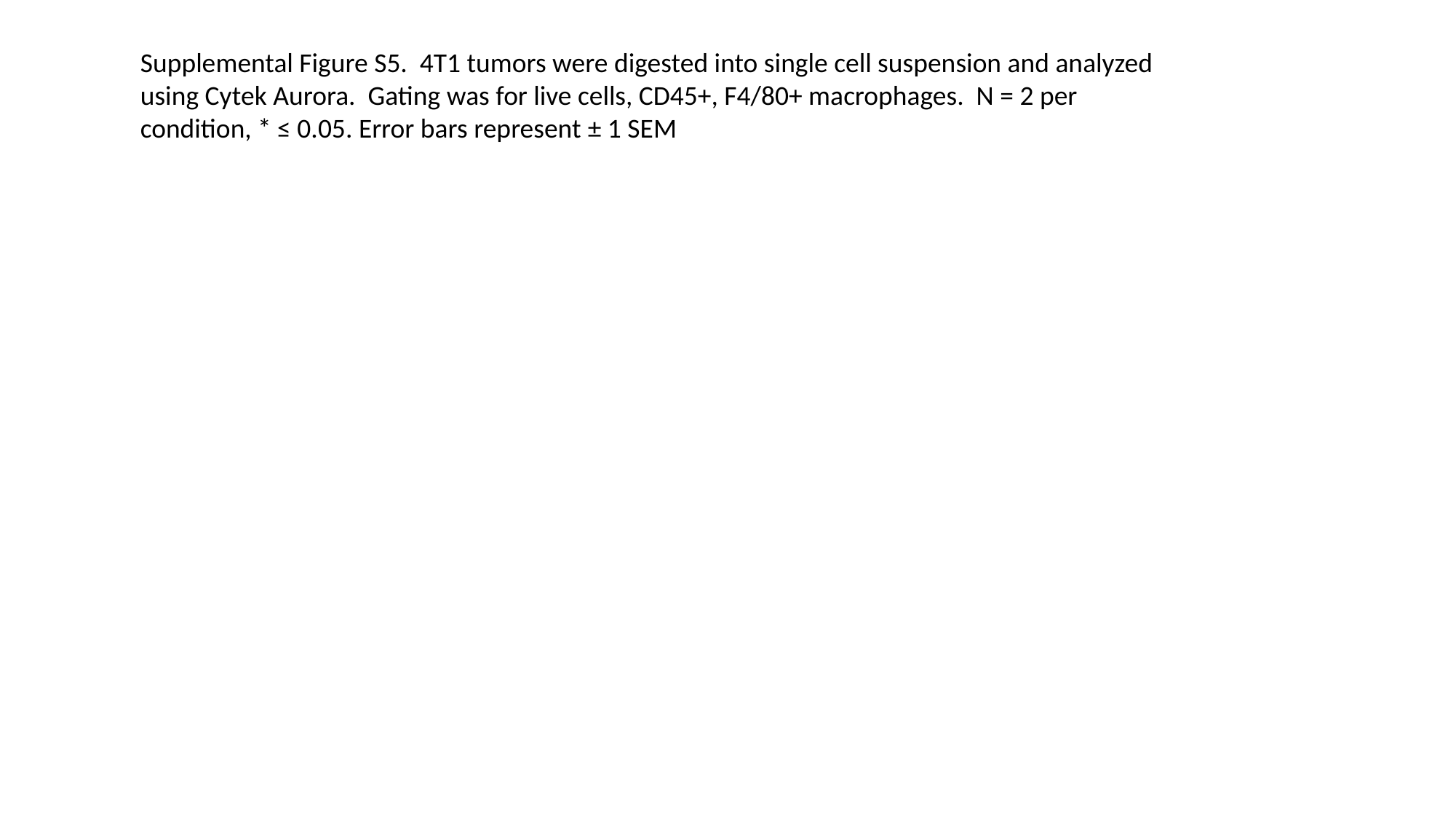

Supplemental Figure S5. 4T1 tumors were digested into single cell suspension and analyzed using Cytek Aurora. Gating was for live cells, CD45+, F4/80+ macrophages. N = 2 per condition, * ≤ 0.05. Error bars represent ± 1 SEM

## Slide 12
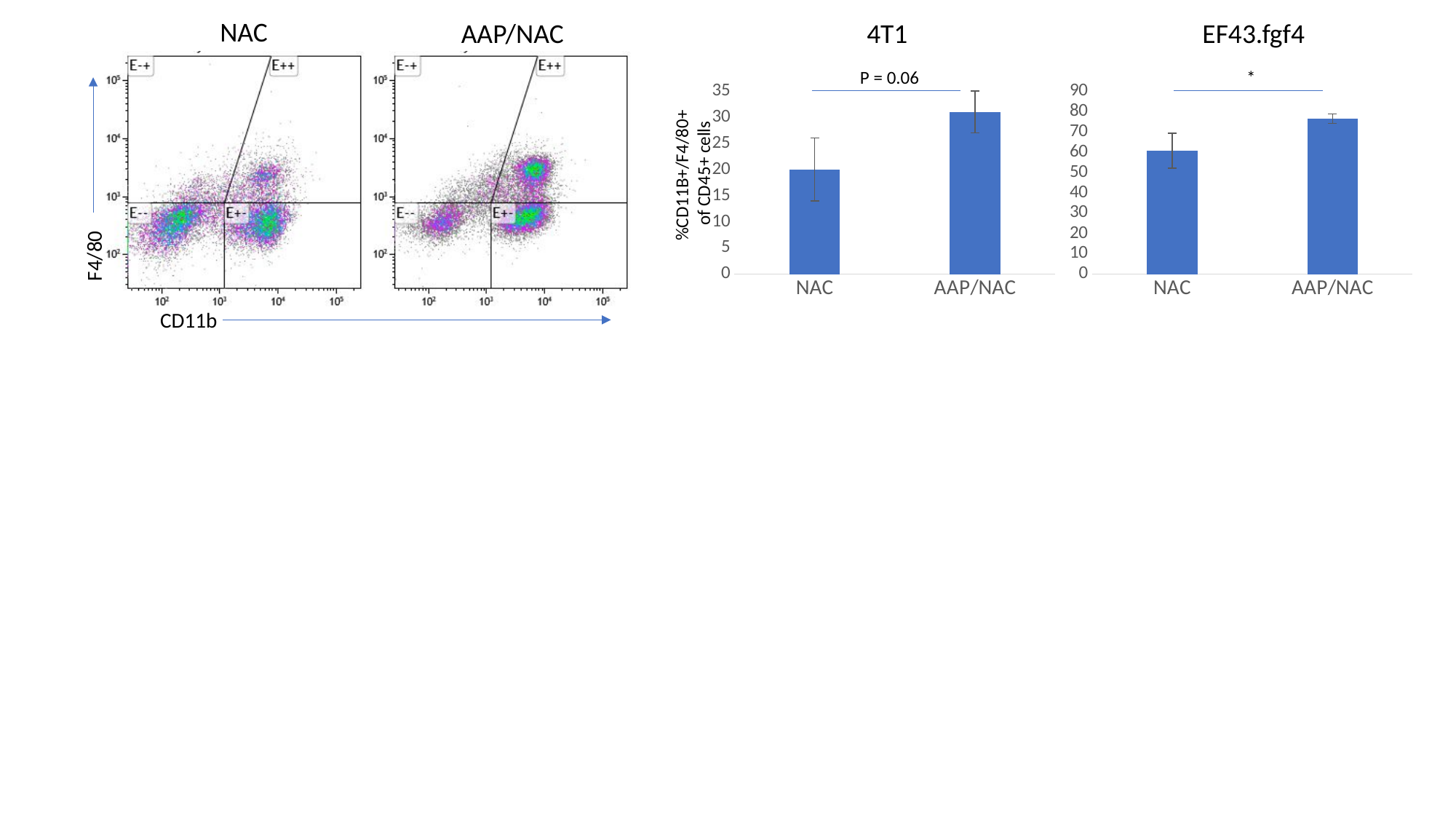

NAC
AAP/NAC
4T1
EF43.fgf4
P = 0.06
*
### Chart
| Category | |
|---|---|
| NAC | 60.666666666666664 |
| AAP/NAC | 76.33333333333333 |
### Chart
| Category | |
|---|---|
| NAC | 20.0 |
| AAP/NAC | 31.0 |%CD11B+/F4/80+
of CD45+ cells
F4/80
CD11b

## Slide 13
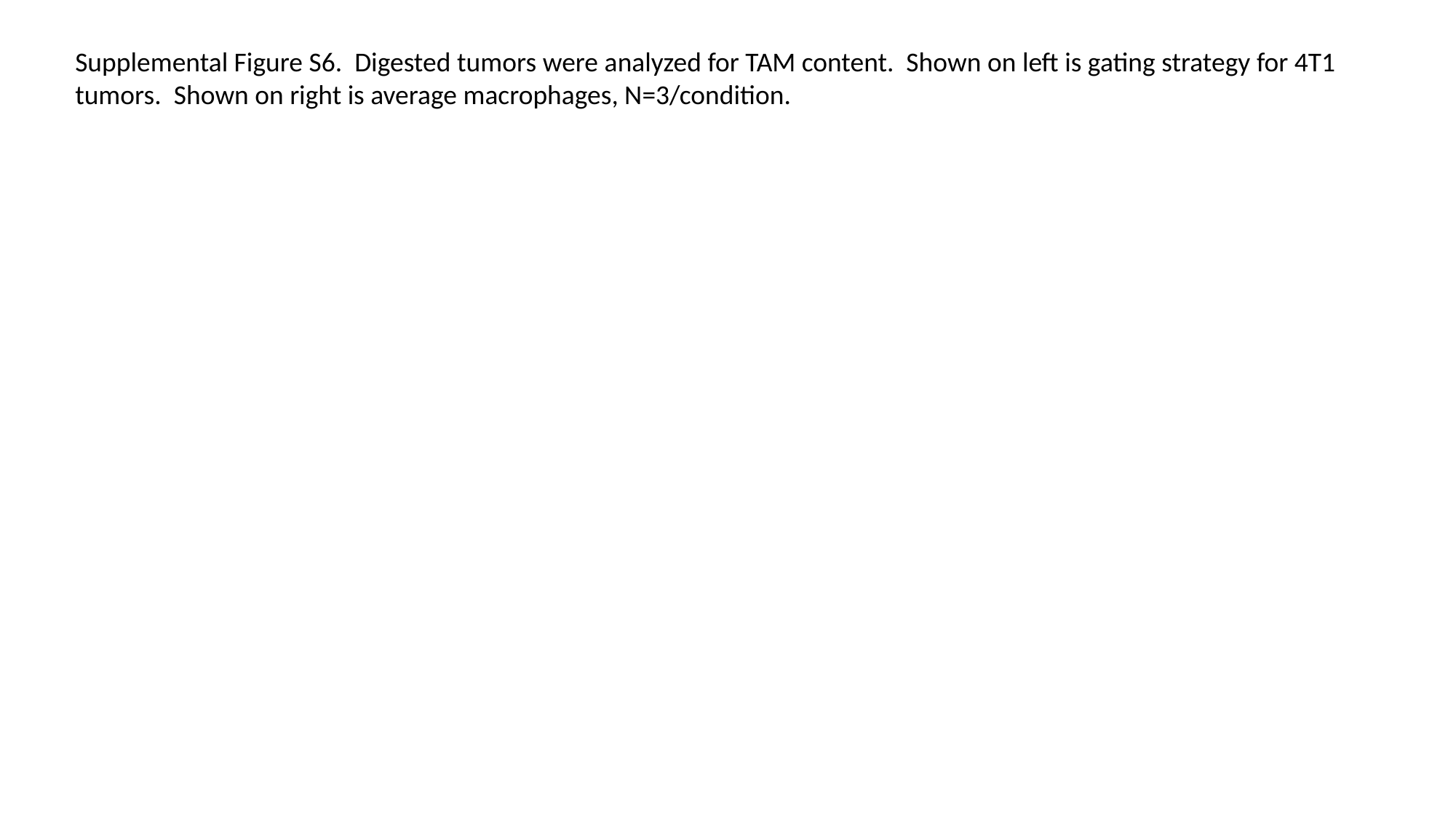

Supplemental Figure S6. Digested tumors were analyzed for TAM content. Shown on left is gating strategy for 4T1 tumors. Shown on right is average macrophages, N=3/condition.

## Slide 14
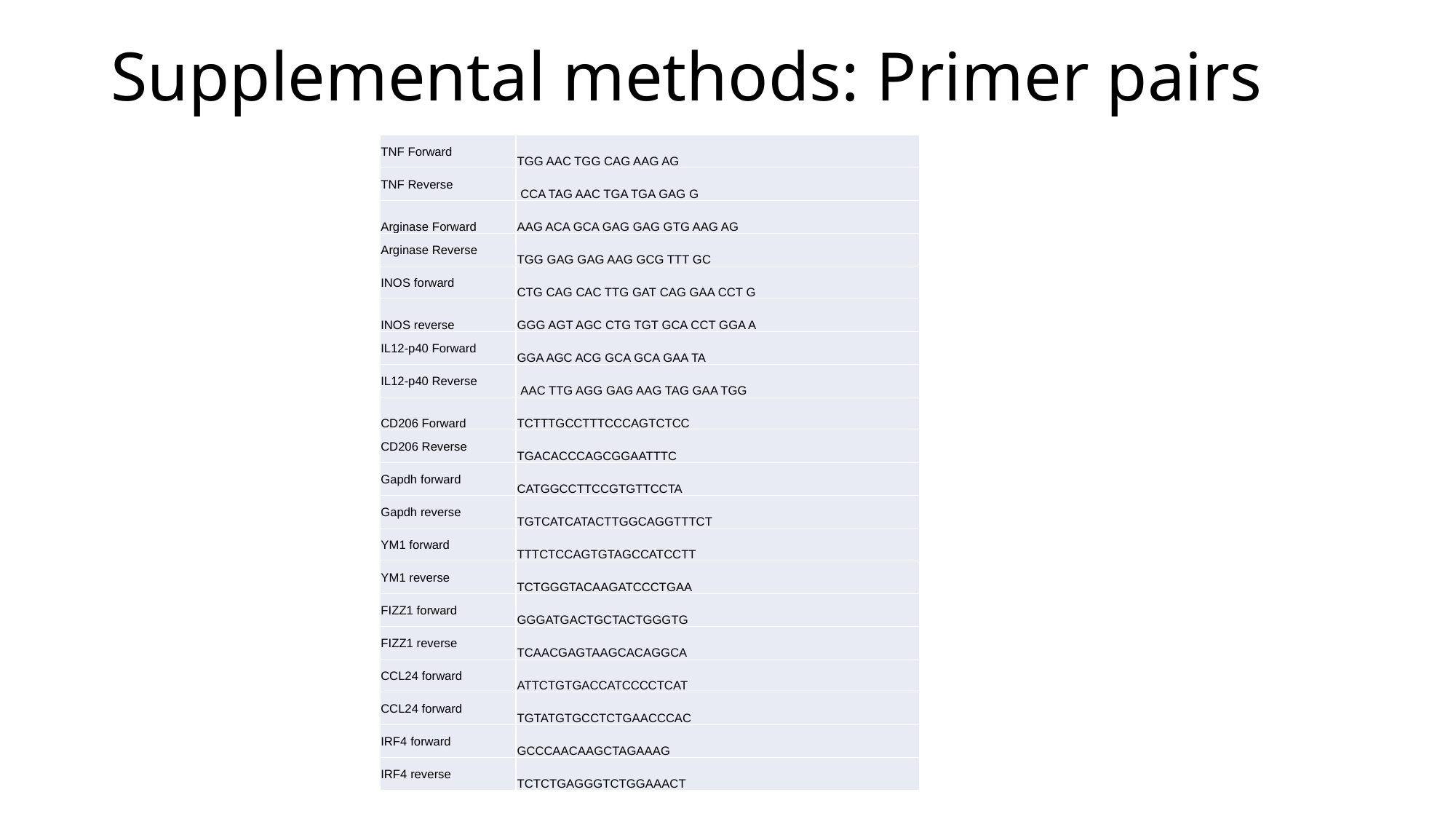

# Supplemental methods: Primer pairs
| TNF Forward | TGG AAC TGG CAG AAG AG |
| --- | --- |
| TNF Reverse | CCA TAG AAC TGA TGA GAG G |
| Arginase Forward | AAG ACA GCA GAG GAG GTG AAG AG |
| Arginase Reverse | TGG GAG GAG AAG GCG TTT GC |
| INOS forward | CTG CAG CAC TTG GAT CAG GAA CCT G |
| INOS reverse | GGG AGT AGC CTG TGT GCA CCT GGA A |
| IL12-p40 Forward | GGA AGC ACG GCA GCA GAA TA |
| IL12-p40 Reverse | AAC TTG AGG GAG AAG TAG GAA TGG |
| CD206 Forward | TCTTTGCCTTTCCCAGTCTCC |
| CD206 Reverse | TGACACCCAGCGGAATTTC |
| Gapdh forward | CATGGCCTTCCGTGTTCCTA |
| Gapdh reverse | TGTCATCATACTTGGCAGGTTTCT |
| YM1 forward | TTTCTCCAGTGTAGCCATCCTT |
| YM1 reverse | TCTGGGTACAAGATCCCTGAA |
| FIZZ1 forward | GGGATGACTGCTACTGGGTG |
| FIZZ1 reverse | TCAACGAGTAAGCACAGGCA |
| CCL24 forward | ATTCTGTGACCATCCCCTCAT |
| CCL24 forward | TGTATGTGCCTCTGAACCCAC |
| IRF4 forward | GCCCAACAAGCTAGAAAG |
| IRF4 reverse | TCTCTGAGGGTCTGGAAACT |
