# Supplementary figures and images for: High-Dose Acetaminophen with N-acetylcysteine Rescue Inhibits M2 Polarization of Tumor-Associated Macrophages
Source: Cancers (Basel). 2023 Sep 28;15(19):4770. doi: 10.3390/cancers15194770 (PMC10571846; doi:10.3390/cancers15194770)

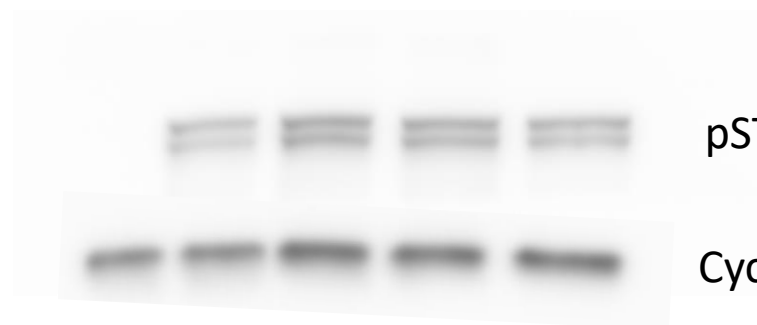

pSTAT1

Cyclophilin

|         |   |   |   |   |   |
|---------|---|---|---|---|---|
| IFN/LPS | - | + | + | + | + |
| AAP     | - | - | - | + | - |
| NAC     | - | - | + | - | + |

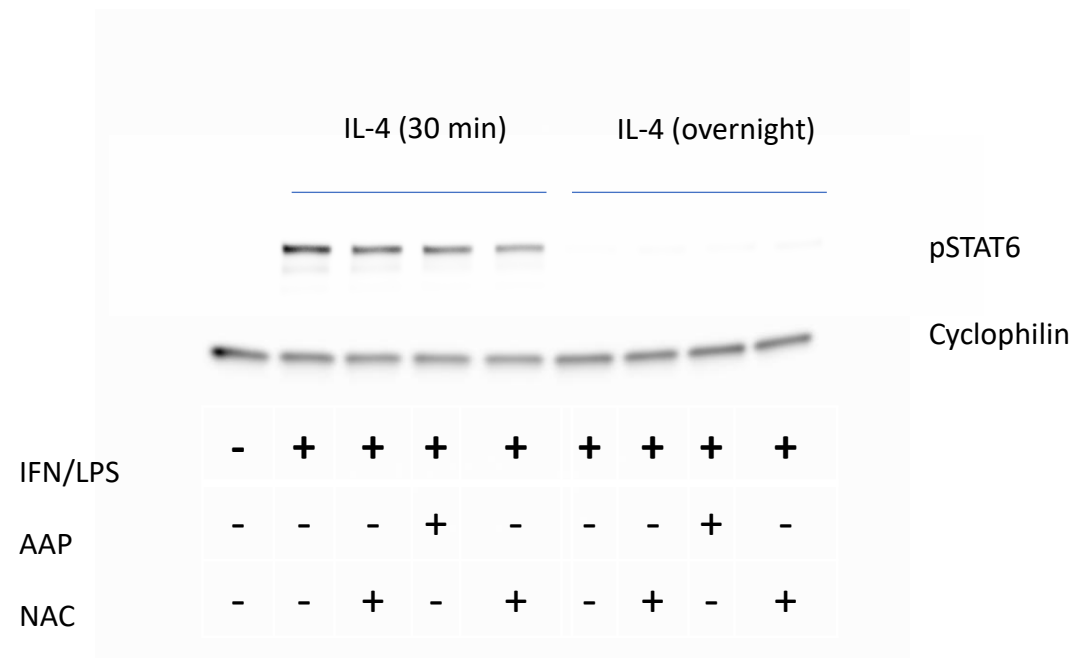

Supplement: Supplementary file 1 [file cancers-15-04770-s001.zip › cancers-2604837-supplementary/cancers-2604837-File S1.pdf]
